# Nail proximal fold stem cells participate in nail growth, orchestrating enhanced digit regeneration via bone morphogenetic protein signaling activation

**DOI:** 10.1093/stmcls/sxag028

**Published:** 2026-04-08

**Authors:** Anna Pulawska-Czub, Alicja Olczak-Cossu, Tomasz D Pieczonka, Krzysztof Kobielak

**Affiliations:** Laboratory of Stem Cells, Development and Tissue Regeneration, Centre of New Technologies, University of Warsaw, Warsaw, 02-097, Poland; Laboratory of Stem Cells, Development and Tissue Regeneration, Centre of New Technologies, University of Warsaw, Warsaw, 02-097, Poland; Laboratory of Stem Cells, Development and Tissue Regeneration, Centre of New Technologies, University of Warsaw, Warsaw, 02-097, Poland; Laboratory of Stem Cells, Development and Tissue Regeneration, Centre of New Technologies, University of Warsaw, Warsaw, 02-097, Poland; Faculty of Medicine, University of Warsaw, Warsaw, 02-089, Poland

**Keywords:** nail proximal fold stem cells, nail mini-organ, digit regeneration, third phalanx bone amputation, BMP and Wnt signaling

## Abstract

Rodent and primate digit tips exhibit a remarkable regenerative capacity following amputation, driven by highly proliferative nail stem cells with active canonical Wnt signaling. Recently, a distinct, slow-cycling population of bi-functional nail proximal fold stem cells (NPFSCs) has been identified, contributing to both peri-nail epidermis and nail plate (NP). Here, we demonstrate that NPFSCs actively participate in nail growth, orchestrating digit regeneration, with bone morphogenetic protein (BMP) signaling serving as a key regulator. Inhibition of BMP resulted in an epidermalized NP-like structure with limited regeneration due to impaired Wnt pathway activation in the nail matrix cells. Conversely, BMP activation enhanced NPFSCs’ involvement in the nail matrix and significantly promoted digit regeneration. We further revealed that enhanced BMP activity not only accelerated nail and bone regrowth but also extended the regenerative boundary proximally, enabling full regeneration after up to ∼60% removal of the distal phalanx (P3). Moreover, in BMP gain-of-function models, extreme proximal amputation, removing the majority of the P3, still permitted partial NP regeneration despite the absence of bone reconstruction. Finally, we isolated and cultured lineage-traced NPFSCs and transplanted them into immunocompromised mice, where they integrated into the nail proximal fold and contributed to nail matrix progenitors during regeneration. Transplanted NPFSCs retained their regenerative capacity *in vivo*, highlighting their therapeutic potential. Collectively, our findings identify a pivotal role of BMP signaling in mediating NPFSC-driven digit regeneration, reveal BMP-Wnt cross-talk as essential to this process, and provide a framework for enhancing regenerative outcomes in previously non-regenerative contexts following traumatic amputation.

## Graphical Abstract

**Graphical Abstract:**
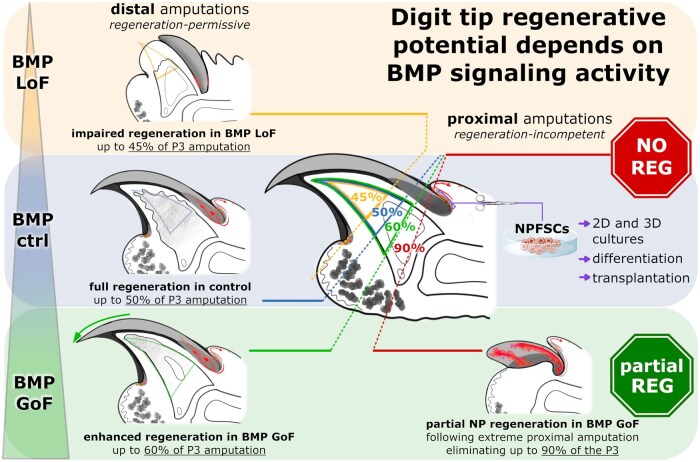

Significance statement We identify nail proximal fold stem cells (NPFSCs) as contributors to nail growth and coordinators of digit tip regeneration after amputation and show that bone morphogenetic protein (BMP) activity governs their fate and regenerative potential. Enhanced BMP signaling accelerates nail and bone regrowth and extends the regenerative boundary, enabling full digit restoration after removal of up to 60% of the distal phalanx. Conversely, BMP ablation impairs Wnt activation in the nail matrix, blocking regeneration, underscoring the key BMP-Wnt interplay. We further demonstrate that isolated and cultured NPFSCs retain their capacity to drive regeneration upon transplantation, highlighting their promising therapeutic potential.

## Introduction

**Figure 1.**
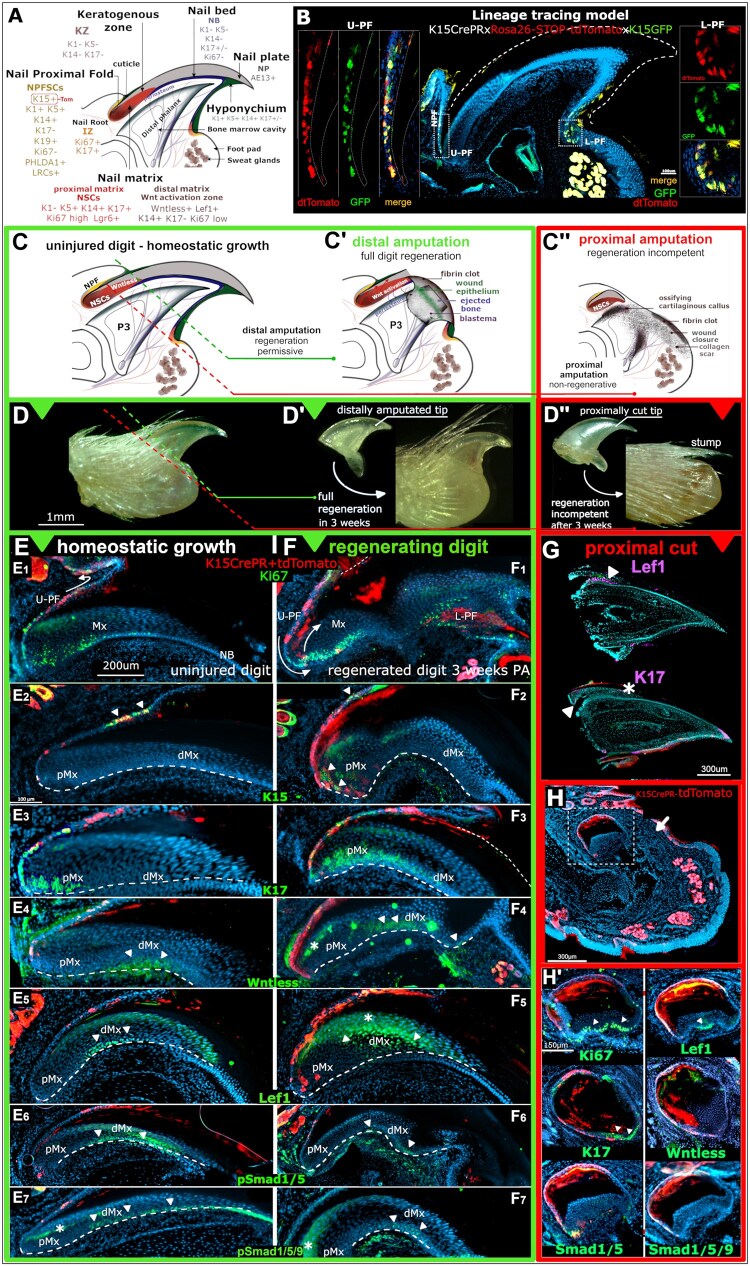
The lineage tracing mouse model K15CrePR/Rosa26-STOP-tdTomato/K15-GFP enables tracking marked NPFSCs throughout nail growth and digit regeneration. (A) The schematic overview of the mouse nail mini-organ with characteristic markers for each structure. (B) The K15-positive NPFSCs labeled with inducible Tom (red) and constitutively expressed GFP (green) are located in the NPF, organized in a ring-like configuration around the nail root; in the dorsal-ventral section, visible as two clusters—above (upper proximal fold, U-PF) and below (lower proximal fold, L-PF) the nail plate. (C) Schematic representation of the digit tip amputation at distal (green dashed line) and proximal (red dashed line) level. (C′) Full digit tip regeneration is only possible after the distal amputation, which eliminates no more than 50% of the distal third phalanx (P3) area, and leaves the distal, Wntless-expressing nail matrix intact. (C″) Proximal amputation leaves the injury site regeneration-incompetent. (D) Image of uninjured mouse digit with dashed lines indicating distal (green) and proximal (red) amputation sites. (D′) Images of distally amputated tip (left) and of fully regenerated digit 3 weeks after distal amputation. (D″) Image of proximally amputated tip (left) and regenerative incompetent stump 3 weeks after proximal amputation. (E_1-6_) The immunofluorescence staining of the naturally grown, uninjured mouse nail mini-organ sections compared to (F_1-6_) sections of the regenerated digit 3 weeks after the distal amputation; pMx–proximal matrix; dMx–distal matrix; dashed line separates nail matrix from the lower dermis; *non-specific signal. (E_1_) Section of uninjured digit in which under normal conditions, the labeled progeny of slow-cycling NPFSCs (red) contribute to the peri-nail epidermis (arrow). (F_1_) Section of the regenerated digit 3 weeks after distal amputation shows NPFSCs (red), additionally providing progeny to the nail matrix and differentiating further into the NP (arrows). (G) Sections of proximally amputated tip with localization of Lef1-positive region, marking the Wnt-activation zone of the nail matrix, and K17-positive proximal matrix (arrowheads); *non-specific signal. (H) Section of regenerative-incompetent stump 3 weeks post proximal amputation, showing dense tissue at the injury site (arrow). (H′) Magnified sections of the nail root sealed in the regeneration-incompetent stump.

Mammalian digit tips can regenerate after amputation; however, this capacity is restricted to the nail-associated region.^1–3^ Mouse nail mini-organ shares key structural features with the human nail unit and thus has become a well-established model for studying nail biology and digit tip regeneration.^4–7^ Two stem cell populations have been identified within the nail unit: highly proliferative nail stem cells (NSCs) in the nail matrix^8^ and a quiescent, slow-cycling nail proximal fold stem cells (NPFSCs) located in the basal layer of the nail proximal fold (NPF) (Figure 1A).^5^^,^^9^ K15-based lineage tracing previously showed that NPFSCs contribute long-term to peri-nail epidermal maintenance and, after injury, can supply progeny to the matrix from where they differentiate into the nail plate (NP).^5^^,^^9^

Digit tip regeneration is a multistep process involving wound closure, blastema formation, and regrowth of the lost structures.^7^^,^^10–15^ In mice, successful regeneration occurs after distal amputations that remove up to 50% of the third distal phalanx (P3) volume, preserving the proximal nail matrix, whereas more proximal amputations fail to support a comparable regenerative response.^16–18^ This regenerative competence depends strongly on the nail organ, including Wnt signaling activity with Wntless-expressing zone in the distal nail matrix, which is required for nail growth and digit regeneration.^8^^,^^19^ However, although the cellular and molecular mechanisms governing blastema formation, bone regrowth, and NSC-dependent regeneration have been extensively studied,^8^^,^^12–18^ the contribution of NPFSCs to nail growth and digit tip regeneration remains unclear.

Recent expression profiling of NPFSCs implicated bone morphogenetic protein (BMP) signaling in nail homeostasis and regeneration.^9^^,^^20^^,^^21^ Consistently, loss of BMP signaling in the nail epithelium was associated with NP abnormalities, loss of the keratogenous zone (KZ), and epidermalization of the nail unit.^9^ In parallel, BMP-related pathways were shown to promote aspects of regeneration after proximal amputations, although these studies did not address either the role of NSCs or NPFSCs populations, nor fully restored the regenerative program.^22–24^ Together, these findings suggested that BMP signaling may regulate NPFSC fate and influence regenerative outcome.

Here, we investigated the role of NPFSCs in nail regrowth and digit tip regeneration after distal and proximal amputations and asked how BMP signaling controls their fate and regenerative potential. We also tested whether NPFSCs can be isolated, expanded *in vitro*, and retain regenerative capacity after *in vivo* transplantation.

## Methods

A summary of the experimental mouse models is provided below, whereas detailed Materials and Methods are available in Supplementary Materials and Methods.

### Mice lines generated

The following mouse lines were generated and used:

**Lineage tracing model; “control model”—**K15CrePR/Rosa26-STOP-tdTomato/K15-GFP^25^; induced with Mifepristone (RU486) enabled detection of K15-expressing NPFSCs and their progeny; K15-GFP enabled detection of NPFSCs in their quiescent stage.

**BMP GoF (gain-of-function) model—**K15CrePR/Rosa26-STOP-tdTomato/K14rtTA/Tre-Alk3Q233^25–27^; the constitutively active form of BMP receptor type A1 was induced under a Doxy-inducible promoter.

**BMP LoF (loss-of-function) model—**K14CreERT/BMPR1A flox/flox^28–30^; specific deletion of the BMP receptor type A1 in the K14-expressing nail epithelium, induced with Tamoxifen (Tam).

## Results

### *In vivo* lineage tracing identifies NPFSC contribution to digit regeneration after permissive distal amputation

To define the role of NPFSCs in nail homeostasis and regeneration, we generated K15CrePR/Rosa26-STOP-tdTomato/K15-GFP mice, in which K15-expressing NPFSCs and their progeny are labeled with Tomato reporter protein after induction by 5-day consecutive Mifepristone (RU486) treatment (Figure 1A and B).^9^^,^^25^^,^^31^ Additional constitutive K15-GFP expression enables identification of NPFSCs in their quiescent state. Shortly after RU induction, Tomato (Tom) expression, largely overlapped with K15-GFP signals, confirming efficient labeling of quiescent NPFSCs in the basal layers of upper and lower NPF (Figures 1B, red subpanel cells in magnification from NPFSCs, on the left for upper NPF (U-PF), and on the right for lower NPF (L-PF)), suggesting their arrangement in a ring-like formation surrounding the nail root. From there they contribute long-term to the peri-nail epidermis (Figure 1E1 arrow), as evidenced by Tom-positive cells in the basal layer of epidermis overlying the upper NPF. In contrast, Ki67-positive proliferating NSCs were concentrated in the proximal matrix (Figure 1E1, green cells in Mx), whereas, as expected, the quiescent, slow-cycling Tom-positive NPFSCs remained negative for Ki67 staining (Figure 1E1). Few Ki67-positive cells were also detected in the NPF area directly above and below the NPFSCs.

The injury model involved amputations at different levels along the NP and the distal third phalanx (P3) (Figure 1C and D and Figure S1A′ [see online supplementary material for a color version of this figure], dashed lines indicate the amputation plane). Complete regeneration occurred only when no more than 50% of P3 was removed, while preserving the entire proximal matrix and a substantial portion of the distal matrix (Figure S1A′ and B—*x* axis marks the % of amputated P3; and S1C blue graph [see online supplementary material for a color version of this figure]). Under these conditions, the regenerating bone reached maximal expansion at ∼5weeks post amputation (PA), acquiring an irregular, still loosely packed bone morphology and periosteal outline, followed by gradual remodeling, restoring native morphology by ∼9 weeks PA (Figure S1A′ [see online supplementary material for a color version of this figure], 5 and 9 weeks, respectively, in right panels). Crucially, regeneration-permissive amputations retained the entire proximal matrix and a substantial portion of the distal matrix, both of which remained embedded within the injured tissue.^8^^,^^18^ Deeper amputations removing a larger portion of P3 resulted in markedly impaired digit tip regeneration progression (Figure S1B [see online supplementary material for a color version of this figure]).

Three weeks after permissive distal amputation (Figure 1C′ and D′ [see online supplementary material for a color version of this figure])—during digit regeneration, the typically quiescent NPFSCs began generating Tom-positive progeny, which migrated to the proximal matrix and subsequently differentiated through the KZ into the direction of the hard NP (Figure 1F1). Keratin 15 (K15) staining was expanded into the proximal matrix and correlated with Tom-labeled descendants (Figure 1F2). In comparison, during nail homeostasis, both K15 staining and Tom-labeled progeny marked NPFSCs predominantly in the NPF (Figure 1E2). Keratin 17 (K17) staining in the uninjured digits was observed largely in the proximal matrix, marking physiologically highly proliferative NSCs (Figure 1E3), while at 3 weeks of regeneration, a stretched population of K17-positive cells was observed in the proximal and distal matrix (Figure 1F3). Wntless and Lef1 (lymphoid enhancer-binding factor 1), markers of Wnt pathway, were detected in the distal matrix in uninjured digits (Figure 1E4 and E_5_, respectively) and remained prominent during regeneration (Figure 1F4 and F_5_, respectively). Alongside this Wnt activation area, in the distal nail matrix, phosphorylated Smad1/5 (pSmad1/5) (Figure 1E6 and F_6_) and phosphorylated Smad1/5/9 (pSmad1/5/9) (Figure 1E7 and 1F_7_) were also present.

**Figure 2.**
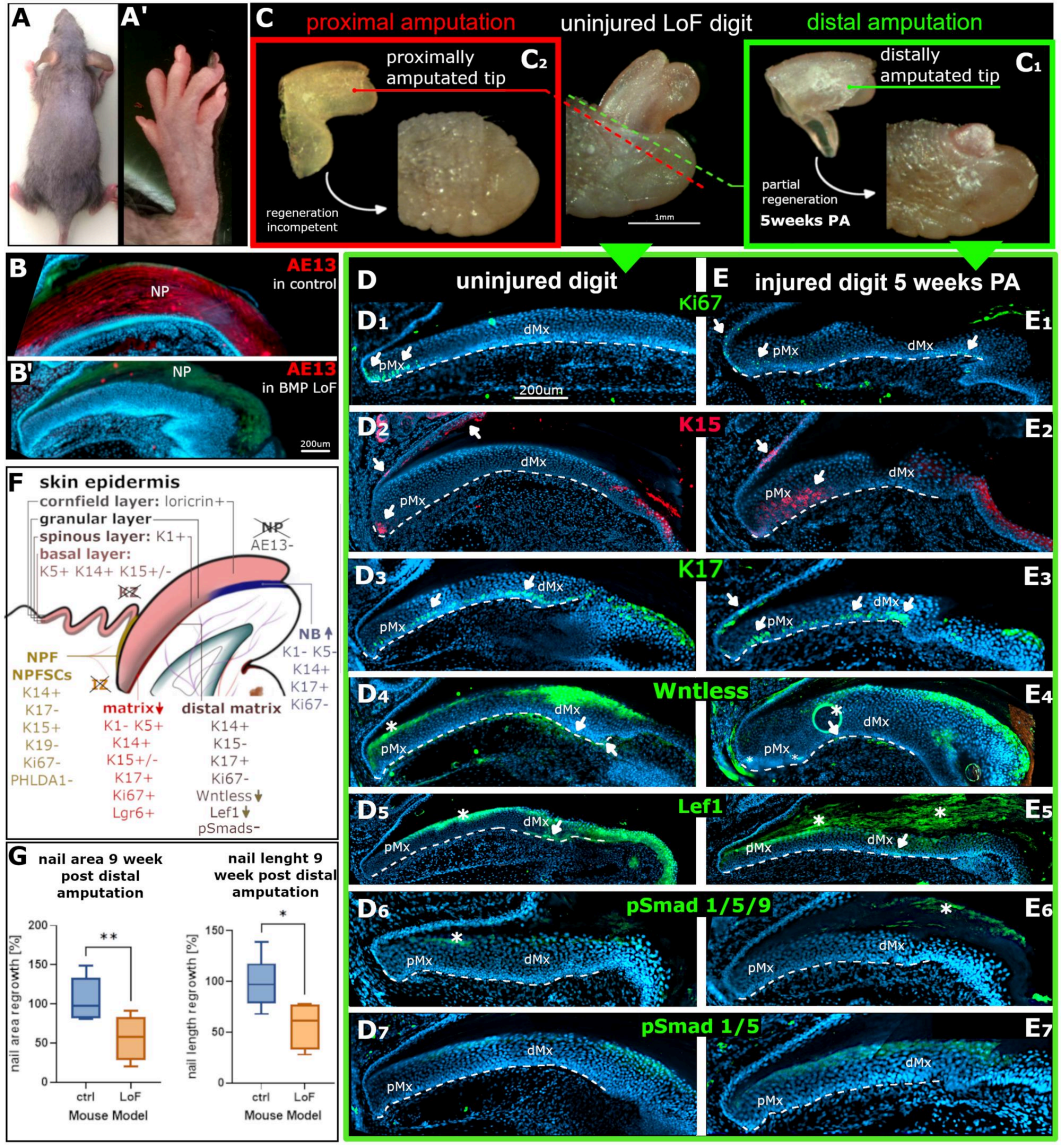
Loss of the BMP signaling inhibits the proper NP differentiation and significantly impairs digit regeneration. (A) Transgenic mouse model LoF K14CreERT/BMPR1A with deleted BMP receptor A1 (Bmpr1a) in K14-positive keratinocytes of the developing skin is associated with loss of hair and abnormal nail structure (A′). (B) AE13 expression (red), characterizing hard keratins in the properly formed NP, is absent in the LoF nail structures (B′). (C) The close-up of the BMP LoF nail phenotype with dashed lines representing levels of amputation (green–distal; reg–proximal). (C_1—_green frame) Impaired digit regeneration 5 weeks after distal amputation. (C_2—_red frame) Lack of regeneration following proximal amputation. (D_1-7_) Immunofluorescence staining of sections of uninjured BMP LoF digits, compared to (E_1-7_) sections of regenerated BMP LoF digits 5 weeks after distal amputation; pMx–proximal matrix; dMx–distal matrix; dashed line separates nail matrix from the lower dermis; *non-specific signal. (F) Schematic overview of the BMP signaling-deprived murine digit. (G) The comparison of regeneration efficiency between the BMP LoF digits (orange) and control (lineage tracing) model (blue), expressed as a percentage of the originally amputated NP length (left plot) or surface area (right plot) restored within 9 weeks after distal amputation. **p < 0.01. *p < 0.1; unpaired *T*-test.

**Figure 3.**
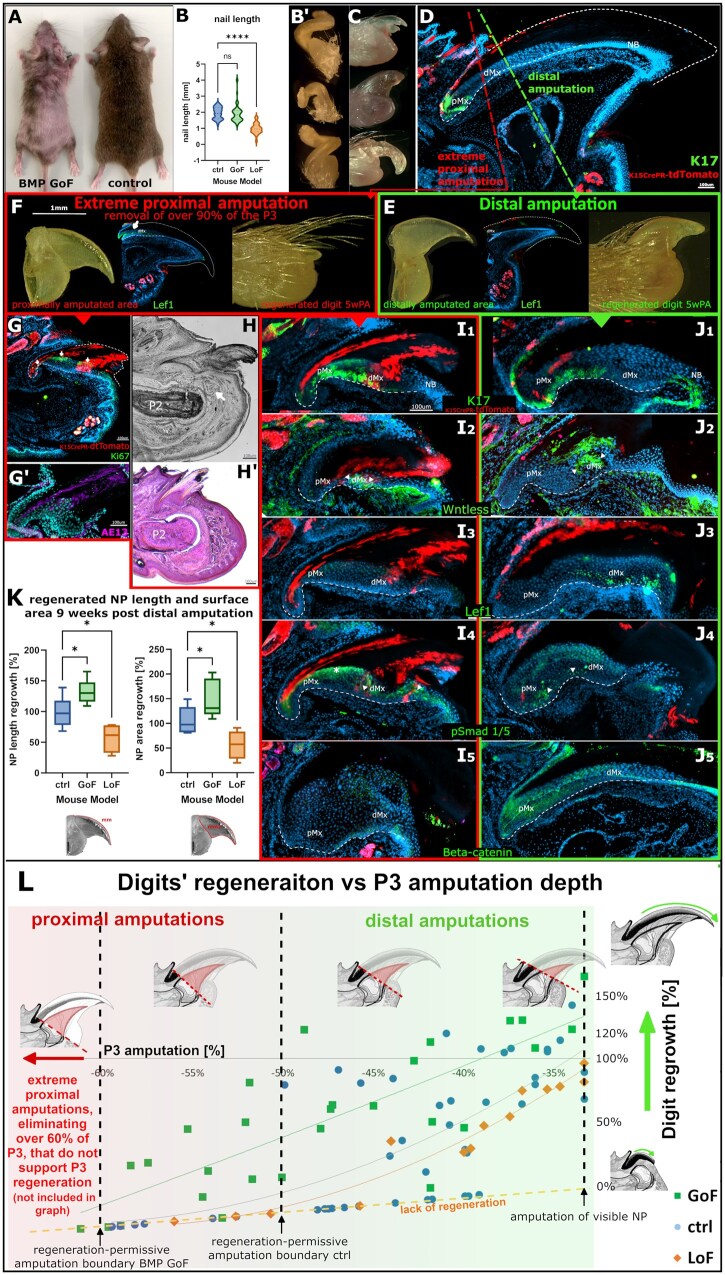
BMP signaling overexpression accelerates digit tip regeneration, expands the regeneration-permissive boundary beyond the proximal amputation limit, and allows partial recovery after extreme proximal resection. (A) Phenotype of K15CrePR/Rosa26-STOP-tdTomato/K14rtTA/Tre-Alk3Q233 BMP GoF mice showing disrupted hair growth. (B) Average nail length and area in BMP GoF mice (green) were comparable to control model (lineage tracing) digits (blue); (B′) Occasional thumbnail overgrowth in aged BMP GoF mice (P > 250). (C) Representative example of abnormal NP overgrowth after tip amputation in BMP GoF model. (D) Cross-section of uninjured BMP GoF nail mini-organ with Tom-positive NPFSCs (red) in the NPF; distal and extreme proximal amputations are marked with green and red dashed lines; K17-expression marks proximal matrix (green). (E) Image of distally amputated tip, covering Lef1-negative proximal matrix, and regeneration outcome at 5 weeks. (F) Image of resected tip following extreme proximal amputation, that removed most of Lef1-positive distal matrix (arrow, green); and regeneration outcome at 5 weeks. (G) Section of regenerating BMP GoF digit at 5 weeks post extreme amputation with Tom-labeled NPFSC progeny (red) contributing to nail root, matrix, and NP; Ki67 staining marks proliferating cells (arrows, green). (G′) AE13 staining (purple) of regenerating BMP GoF digit at 5 weeks post extreme amputation. (H, H′) DIC and H&E staining shows lack of P3 renewal 5 weeks post extreme proximal amputation. (I_1-5_) IF stained sections of incompletely regenerated BMP GoF digit at 5 weeks post extreme proximal amputation compared to fully regenerated digits following distal amputation (J_1-5_); pMx–proximal matrix; dMx–distal matrix; dashed lines indicate nail matrix–dermis boundary; *non-specific signal. (K) NP regeneration after distal amputation quantified at 9 weeks shows the highest efficiency in BMP GoF (green), lowest in BMP LoF (orange), vs. controls (blue—lineage tracing); *P < .1. (L) Graph showing a percentage of digit regeneration (y-axis) vs. P3 amputation depth (x-axis); red background marks more proximal amputations, green–distal. Linear trends indicate regeneration potential in BMP GoF (green, squares), BMP LoF (orange, diamonds), and control (lineage tracing) model (blue circles) mice. Regeneration in control (lineage tracing) model digits is observed only when less than 50% of P3 is amputated (dashed vertical line at 50% on the *x*-axis), whereas in BMP GoF digits, regeneration extends up to amputations removing nearly 60% of the P3 (dashed vertical line at 60% *x*-axis).

**Figure 4.**
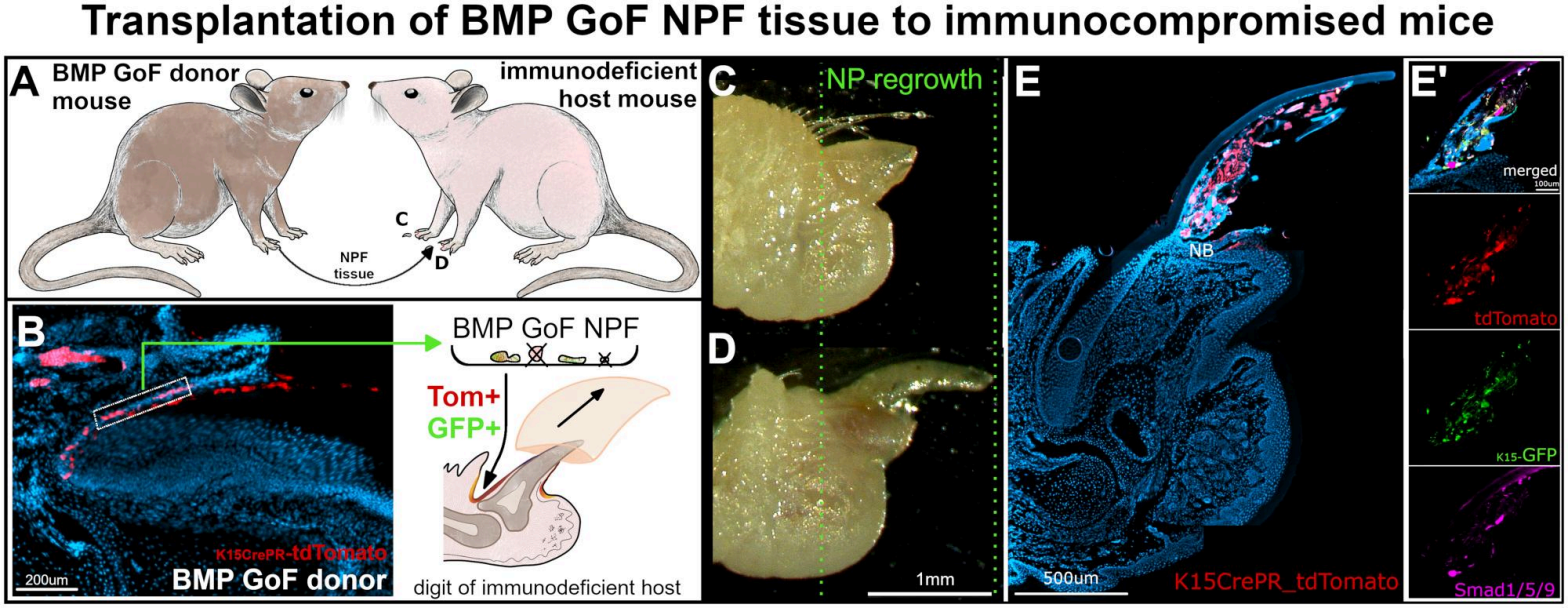
The NPF tissue collected from the BMP GoF mouse model with overexpressed BMP signaling participates in nail mini-organ reconstruction after transplantation. (A) The BMP GoF mice with an additional K15-GFP reporter gene were used to collect the NPF tissue, which was then transplanted into the NPF pocket created by plucking out nails of the immunocompromised mice. (B) Only tissues harboring dually labeled BMP GoF NPFSCs (Tom+ red and GFP+ green) were used as grafts. (C) Nail that regrown following its removal by plucking was half the length of nail that was additionally subsequently transplanted with BMP GoF NPF tissue (D). (E) Cross-sectioning with the positioning of the Tom-pos graft (red—NPFSC progeny) in the restored nail bed structure. (E′) IF staining of sections containing transplanted tissue: K15-GFP (green), pSmad 1/5/9 (purple).

By contrast, proximal amputations (Figure 1C″ and D″), which removed the entire distal part of nail matrix (Figure 1G), resulted in loss of regenerative capacity which correlated with impaired contribution of Tom-positive NPFSCs progeny in NP (Figure 1D″ and H). The sections of the amputated fragment revealed that majority of the Lef1-expressing area had been eliminated (which reflects Wntless expression), and the cut interfered with the terminal part of the K17-positive proximal matrix (Figure 1G). While reduced Lef1 expression partially reappeared in the subcutaneous portion of the remaining nail mini-organ, the Wntless-expressing region was absent in basal proximal matrix, which made it impossible for the lingering part of nail proximal matrix to regrow and emerge from the overlying healed tissue, even though the expression of Ki67 and K17 was still present in trapped proximal matrix (Figure 1H and H′). Moreover, these regeneration-incompetent stumps exhibited no expression of both pSmad1/5/9 and pSmad1/5 for canonical BMP signaling in nail proximal matrix, which correlated with the absence of Wntless-positive nail matrix, highlighting a critical role of the BMP signaling in facilitating regenerative response (Figure 1H′).

### Loss of BMP signaling in the nail epithelium impairs nail and digit tip regeneration

To investigate the role of BMP signaling in nail and digit regeneration, we specifically deleted the BMP receptor type A1 (*Bmpr1a*) in developing nail mini-organs using a K14Cre-ERT-mediated approach, thus generated BMP LoF mouse model.^30^^,^^32^^,^^33^ As in this model the ablation of BMP signaling targets all K14-expressing epithelial cells, it was used to assesses the global impact of epithelial BMP depletion on regenerative outcome *in vivo* rather than NPFSC-specific BMP function, and therefore, the contribution of these cells was not directly assessed in this particular LoF model. Instead, the specific contribution of NPFSCs was addressed separately using lineage tracing (described in the above section) and BMP GoF models (described in the following section) in functional regeneration assays.

Conditional BMP LoF mutations were induced by administering Tamoxifen to pups from postnatal day 2 to day 6. Although the treatment was applied locally to the paws, the observed BMP LoF phenotype was systemic and manifested clearly in the skin as extreme hair loss (Figure 2A), which correlated with the phenotype of the mice following BMPR1A ablation in skin and hair described previously.^34^ Loss of BMP signaling disrupted a proper KZ formation and NP differentiation (Figure 2A′), as AE13-positive hard NP keratins present in lineage tracing mice were absent (Figure 2B vs. B′), and instead, the appendage was covered with a granular layer resembling extended skin epidermis, as previously reported by Leung *et al.*^9^ These skin-like structure coated nails grew more vertically (Figure 2A′ and C) compared to the horizontally extended, claw-shaped nails seen in lineage tracing mice (Figure 1D), and the BMP LoF P3 was visibly shorter (Figure S1A‴ vs. lineage tracing S1A′).

Following distal amputation, BMP LoF digits showed severely impaired regeneration at 5 weeks PA (Figure 2C1). By week 9 PA, the regenerated NP remained significantly reduced in both length and area relative to the original amputated NP (Figure 2G, orange plot), and no P3 regeneration was observed (Figure S1A‴). In contrast, in control lineage tracing mice, the first signs of complete NP regeneration were visible as early as 3 weeks PA after distal amputation (Figure 1D′). By 5 weeks PA, the regenerating P3 had already reached the length of uninjured digit, although its structure remained broad and loosely organized, and full remodeling to its native morphology was achieved only by approximately 9 weeks PA (Figure S1A′). At this stage, the regenerated NP had restored its original length and area on average to 100%, often exhibiting overgrowth (Figure 2G, blue plot).

Impaired regeneration in BMP LoF mice correlated with a marked reduction in Ki67-positive cells in the proximal matrix (Figure 2E1), expansion of K15 staining into the proximal matrix (Figure 2E2), and further extension of the K17-positive domain by 5 weeks PA (Figure 2E3) when compared with uninjured LoF mice (Figures 2D1-3). Wntless and Lef1 expression were strongly reduced in both uninjured and regenerating BMP LoF digits compared with lineage tracing (Figure 2D4,5 and E4,5 vs. Figure 1E4,5 and F4,5). Loss of pSmad 1/5/9 (Figure 2D6 and E_6_) and pSmad1/5 (Figure 2D7 and E_7_) confirmed effective BMP pathway inactivation in the entire nail matrix epithelium when compared to control lineage tracing models (Figure 1E6,7 and F_6,7_).

**Figure 5.**
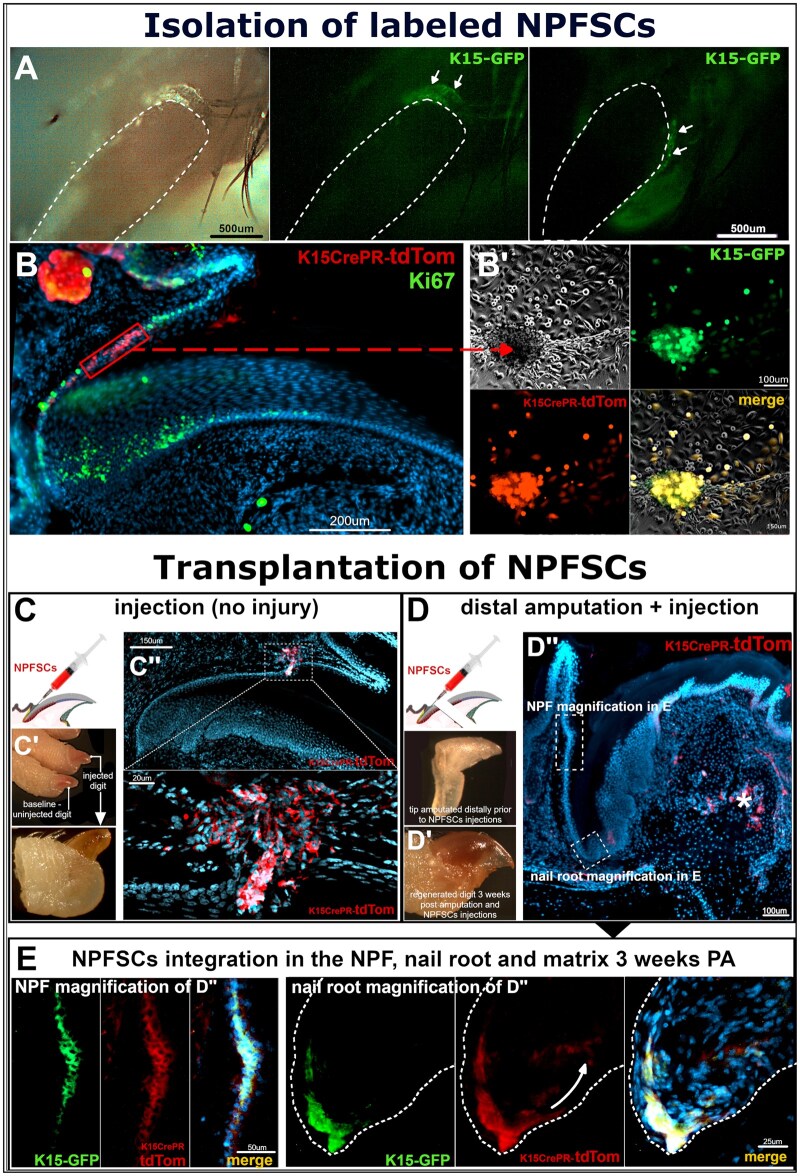
Isolated and cultured labeled NPFSCs integrate into the nail epithelium after transplantation and contribute to regeneration of the injured digit. (A) The upper nail epidermis covering the base of the NP was tilted back to expose the location of the K15-GFP-positive quiescent NPFSCs within the NPF (arrows). (B) Nail section showing the localization of Tom-positive NPFSCs (red) in the lineage tracing mouse model; (B′) Tom-positive NPF tissue was isolated using fluorescence-guided microdissection. Cells co-expressing green and red fluorescent proteins were isolated and expanded in culture and flow cytometry was used to confirm the population homogeneity, demonstrating uniform Tom positivity. (C) Cultured NPFSCs were engrafted underneath the epidermis covering the nail unit of immunocompromised mouse. (C′) 3 weeks after NPFSCs transplantation, the treated digit showed similar morphology to the adjacent non-transplanted digit. (C″) Injected cells (red) were detected in close proximity to the NPF of the treated digit (magnified inset below). (D) Cultured NPFSCs were engrafted underneath the epidermis above the distally amputated digit of immunocompromised mouse. (D′) Regenerated digit at 3 weeks after distal amputation and NPFSCs transplantation. (D″) Section of digit shown in D′ with indicated regions corresponding to the magnified insets below in E; the asterisk marks NPFSC progeny at the regeneration tip. (E) Higher-magnification view of the region shown in D″ demonstrating injected NPFSCs within the NPF and the nail root, with the Tom-positive progeny contributing to the proximal matrix (arrow).

**Figure 6.**
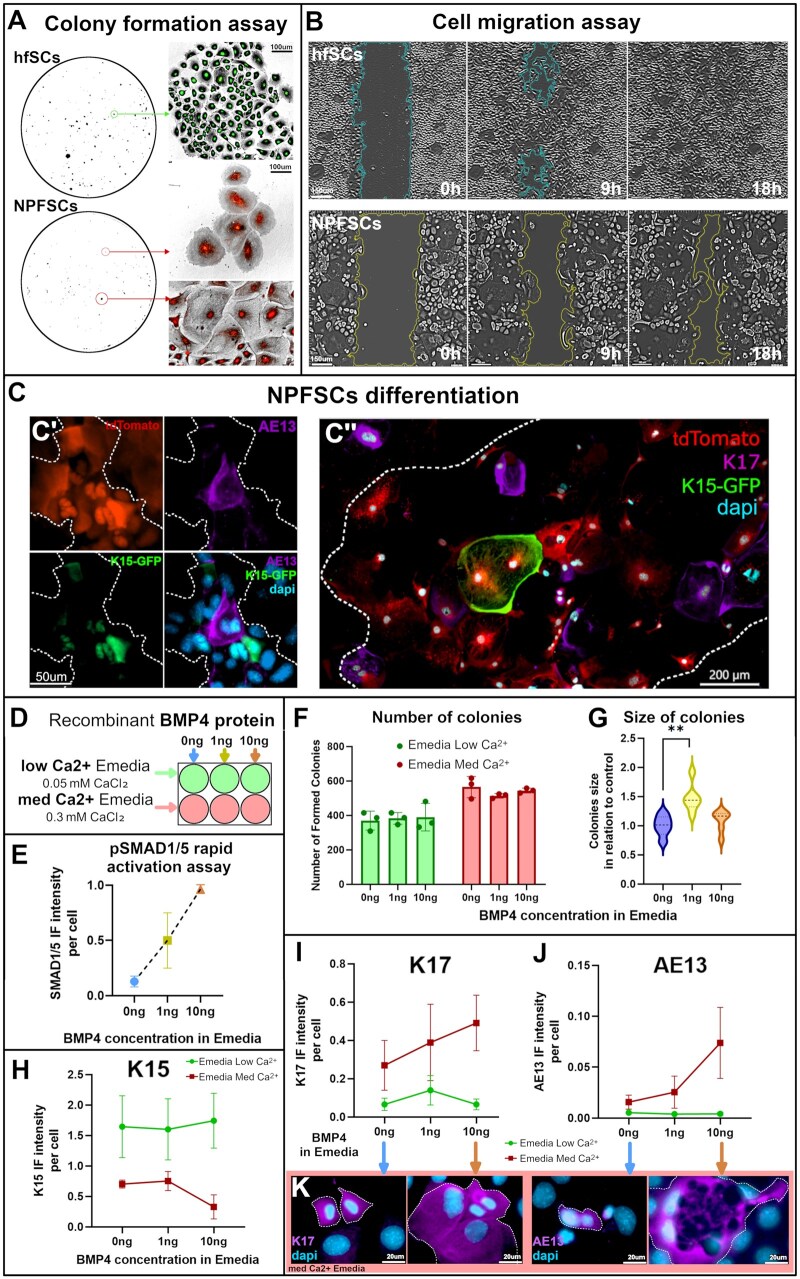
*In vitro* characterization of NPFSCs reveals BMP-dependent differentiation and functional properties in 2D and 3D culture systems. (A) Colony-forming capacity of two skin stem cell populations, hfSCs and NPFSCs. (B) Scratch assay comparing the migratory potential of hfSCs and NPFSCs. (C) Differentiation potential of NPFSCs in 2D and 3D cultures: (C′) IF staining for hard keratins AE13 in 2D cultures, induced to differentiate by elevated Ca2+ concentration in media. (C″) Spheroids generated on Grid3D^®^ 96-well plates stained for K17 at 13 days post-seeding. (D) Schematic overview of NPFSC culture conditions used in the BMP4 enrichment experiment. Cells were cultured in low- or medium-calcium media supplemented with 0, 1 or 10 ng/µL of BMP4 recombinant protein. (E) Rapid pSMAD activation assay at 2 h of BMP4 supplementation (0 ng/µL—blue; 1 ng/µL—yellow; 10 ng/µL—orange), calculated as pSMAD 1/5 IF intensity per cell. (F, G) Colony formation assay across different culture conditions (low Ca2+—green; medium Ca2+—red; and BMP4 concentration—x axis) represented as numbers and sizes of colonies formed. (H-J) IF staining for K15, K17, and AE13, presented as signal intensity per cell across all conditions (low Ca2+—green; medium Ca2+—red, and BMP4 concentration—x axis). (K) Representative 20x magnification images of cells cultured in medium-calcium media IF stained with differentiation markers K17 or AE13 in the absence (0 ng/µL blue arrow) or presence (10 ng/µL orange arrow) of BMP4.

Proximal amputations performed on BMP LoF model were fully non-regenerative (Figure 2C2), similarly as observed in proximal injury in the lineage tracing control model (Figure 1D″).

### Overexpression of BMP signaling in nail epithelium enhances digit regeneration via active engagement of NPFSCs, extends regenerative boundary, and enables partial regeneration following proximal amputation

In opposition to the BMP LoF model, we have engineered a BMP GoF mouse line using double-transgenic (dTg) mice with overexpression of the constitutively active form of BMP receptor type A1 (Bmpr1a-CA or Alk3Q233D) under a doxycycline (Doxy) inducible promoter TRE-Alk3Q233D.^27^^,^^34^ Subsequently, these dTg mice (K14rtTA/Tre-Alk3Q233) were crossed with the background of K15CrePR/Rosa26-STOP-tdTomato, allowing both labeling of NPFSCs and activation of inducible epithelial BMP.^9^^,^^25^^,^^31^ BMP GoF mice placed on a Doxy-supplemented diet displayed severe skin phenotype, resulting in visible hair loss after postnatal day 30 (Figure 3A) as previously published by Kandyba *et al.*,^27^ while uninjured nail growth was broadly comparable to control lineage tracing models, with only occasional thumbnail overgrowth in animals older than 250 postnatal days (Figure 3B and B′). A significantly higher incidence of NP overgrowth was noted in regenerating digits at 5 weeks post distal amputation (Figure 3C). The sections of uninjured BMP GoF nail mini-organs were highly similar to those observed in lineage tracing control models sections with clearly labeled NPFSCs by Tom in the NPF (Figure 3D vs. Figure 1B and Figure S2 [see online supplementary material for a color version of this figure] blue background).

We have investigated how BMP overexpression affects nail and digit regeneration after distal (Figure 3E) and extreme proximal amputation (Figure 3F) as marked schematically by green and red dotted lines on Figure 3D. A distal amputation (Figure 3E, left), which removed half of the P3 (Figure S1A″ [see online supplementary material for a color version of this figure]) and a part of the digits’ Lef1-negative distal matrix (Figure 3E, middle), resulted in full regeneration within less than 3 weeks (Figure 3E, right–digit shown at 5 weeks PA for proper comparison to Figure 3F sections). Moreover, regenerated BMP GoF digits exhibited significantly increased NP length and width compared to control lineage tracing models (Figure 3K). Also, the resected P3 was observed to fully regenerate and regain its native morphology within just 5 weeks PA (Figure S1A″ [see online supplementary material for a color version of this figure]), significantly faster than in lineage tracing control model (Figure S1A′ [see online supplementary material for a color version of this figure]). Surprisingly, although slower, similar regenerative potential (both for NP and P3) was observed for BMP GoF digits that underwent resection exceeding the previously established 50% P3 volume boundary (Figure S1C [see online supplementary material for a color version of this figure] blue graph for regeneration-permissive amputations in lineage tracing control models digits vs. green graph for BMP GoF model, and Figure 3L). Instead, the most extensive amputation that still ensured full digit regeneration in BMP GoF eliminated up to 60% of the P3 (Figure S1C [see online supplementary material for a color version of this figure], green graph). The sections of these regenerating digits displayed major structural similarities to those in regenerating lineage tracing models (Figure 1F and Figure S2 [see online supplementary material for a color version of this figure] green background). Although, as expected, an overexpression of BMP signaling increased levels of pSmad 1/5 and pSmad 1/5/9 in the entire nail matrix, including the proximal part (Figure 3J4 and Figure S2D_3,4,7,8_ [see online supplementary material for a color version of this figure]), which is different from the expression of pSmad1/5 and pSmad1/5/9 observed exclusively in the distal nail matrix of uninjured lineage tracing control mice during their homeostatic growth (Figure 1E6,7 and Figure S2D_1,5_ [see online supplementary material for a color version of this figure]) or regeneration following distal amputation (Figure 1F6,7 and Figure S2D_2,6_ [see online supplementary material for a color version of this figure]).

Remarkably, extreme proximal amputations, performed on BMP GoF models beyond this newly established 60% regeneration-permissive boundary (Figure 3F, left), which would remove up to 90% of P3 (Figure S1D [see online supplementary material for a color version of this figure]) and most of the distal, Lef1-positive matrix (Figure 3F middle), were observed to still support NP regrowth (Figure 3F right panel), although precluding P3 restoration (Figure 3H and H′ and Figure S1D [see online supplementary material for a color version of this figure]). In these partially regenerated digits, Tom-positive NPFSC progeny contributed extensively to the regenerating Ki67-positive nail matrix, from where they fully differentiated into a AE13-positive hard nail structure (Figure 3G and G′, respectively). The newly formed hard structure, which has emerged from the sealed stump and continued to elongate regardless of the lack of P3 beneath it, was shorter and thinner (Figure 3F right panel, 3H arrow marking remnant piece of P3, 3H′ and Figure S1D [see online supplementary material for a color version of this figure]) than the original NP, with its root taking a horseshoe-like shape (Figure 3G and I). K17 expression extended throughout the regenerating matrix following both distal (Figure 3J1) and extreme proximal amputation (Figure 3I1). In the latter case, however, the expanded K17-positive domain contained markedly more Tom-positive NPFSC progeny, which accumulated most prominently at its distal end, where they further differentiated toward the regenerating NP (Figure 3I1, red streaks). In this same distal matrix area, the Wntless expression was retained (Figure 3I2), similarly to the digit restored after distal amputation (Figure 3J2 and Figure S2C_8_ [see online supplementary material for a color version of this figure]); however, Lef1, which was always observed to accompany Wntless (Figures 1E5 and F5 and 3J3, and Figure S2C_1-4_ [see online supplementary material for a color version of this figure]), was notably absent in the matrix of this partially restored, proximally amputated BMP GoF digit (Figure 3I3). Simultaneously, strong β-catenin signal observed in distally amputated regenerating BMP GoF matrix (Figure 3J5) was also visibly reduced in partially regenerated matrix following the deeper cut (Figure 3I5). Lastly, the pSmad1/5 expression was elevated, as in all BMP GoF models (Figure 3J4 in comparison to pSmads expression in control lineage tracing Figure 1F6); however, during partial regeneration following extreme proximal amputation, this signal was mostly concentrated at the distal part of the regenerating tip, strongly associated with labeled NPFSC progeny in the distal matrix (Figure 3I4).

### Transplanted BMP GoF NPF tissue enhances nail regrowth in immunodeficient hosts

NPF tissue was harvested from BMP GoF mouse model, which additionally to K15CrePR/Rosa26-STOP-tdTomato lineage tracing background carried an extra reporter gene responsible for a constitutive expression of K15-GFP, which enabled the localization of NPFSCs in their quiescent state. Using a fluorescent-guided microdissection microscope, tissue fragments containing double-labeled Tom-positive and K15-GFP NPFSCs were selected for transplantation into the digits of immunodeficient NUDE mice (Figure 4A and B). Prior to transplantation, recipient mice were placed on a Doxy-enriched diet, and their NPs were removed by plucking—creating a pocket beneath the NPF for tissue implantation (Figure 4B, schematic). Three weeks after transplantation, the regenerated NP of the engrafted digit displayed approximately twice the length of the spontaneously regenerated adjacent nail, that was similarly removed by plucking, but not engrafted with the NPF tissue (Figure 4D vs. C, respectively). Remarkably, the transplanted NPF tissue with Tom-labeled NPFSCs and their progeny was found integrated into the nail bed of the regenerated mini-organ, from where it contributed to further NP elongation. No labeled cells were detected in the recipient’s native NPF or matrix (Figure 4E). Among the Tom-positive progeny of transplanted NPFSCs (Figure 4E′, red), the K15-GFP was also detected (Figure 4E′, green). Additionally, immunostaining for pSmad1/5/9 confirmed enhanced BMP signaling within the transplanted BMP GoF tissue (Figure 4E′, purple).

### Isolation and expansion of NPFSCs from adult nail mini-organ

To establish NPFSC culture, NPF tissue was microdissected from the digit of lineage tracing mouse model K15CrePR/Rosa26-STOP-tdTomato/K15-GFP and dissociated for growth in cell-supportive conditions (Figure 5A, B, and B′). Cells co-expressing green (GFP) and red (Tom) fluorescent proteins were selected and expanded, generating a purified NPFSC population, which homogeneity was subsequently confirmed by flow cytometry, demonstrating uniform Tom positivity (Figure S3A and B [see online supplementary material for a color version of this figure], red). Interestingly, the majority of cultured cells lost their GFP expression by the second passage, suggesting that K15-GFP gene reporter activity is not maintained *in vitro* as NPFSCs exit quiescence and transition to a more proliferative state (Figure S3A and B [see online supplementary material for a color version of this figure]—reduced green GFP signal compared to strong baseline expression of GFP in Figure 5B′).

### Cultured NPFSCs integrate into nail epithelium after transplantation

To assess behavior after transplantation, cultured Tom-positive NPFSCs were injected into the subcutaneous NPF pocket of uninjured or distally amputated digits of an immunocompromised mouse (Figure 5C and D). The procedure performed on uninjured digits resulted in no visible changes in this nail morphology or growth, compared to adjacent, non-transplanted digits (Figure 5C′). However, the cluster of transplanted Tom-positive cells was discovered at the injection site, integrated in close proximity to the native NPF niche, from where they were not observed to spread to other compartments of the digit (Figure 5C″, red cluster, magnified below). In contrast, when Tom-labeled NPFSCs were injected into digits that had undergone distal amputation shortly prior to transplantation (Figure 5D), they were detected within 3 weeks in both the NPF and nail root, from where their progeny contributed to the distal matrix (Figure 5E[mdash]magnified insets from Figure 5D; left panel—NPF; right panel—nail root; arrow—NPFSCs contribution to nail matrix). The regenerated NP exhibited an unusually enlarged morphology (Figure 5D′); however, the P3 beneath was still undergoing structural remodeling and osteogenic processes following histolysis, suggesting the ongoing reshaping process of the whole tip. Strikingly, within this reorganizing extracellular nail matrix, Tom-positive progeny was also observed deep within the central region of the regenerating tip (Figure 5D″, asterisk). However, further research needs to be performed to determine whether their presence within the tip of regenerating digit reflects functional integration or whether the cells were passively retained during wound closure and tissue remodeling.

### Cultured NPFSCs exhibit proliferative and differentiation potential *in vitro*

To compare NPFSCs with another epithelial stem cell population, we analyzed colony formation and migration relative to hair follicle stem cells (hfSCs). NPFSCs formed fewer colonies and showed lower migratory capacity than hfSCs during a scratch wound healing assay (Figure 6A and B, respectively).

The differentiation potential of cultured NPFSCs was assessed under several culture conditions (Figure 6C–J and Figure S3 [see online supplementary material for a color version of this figure]). In 2D assays, NPFSCs were supplemented with different calcium (Ca^2+^) concentrations (Figure S3 [see online supplementary material for a color version of this figure]). As expected, in low-calcium media (0.05 mM Ca^2+^), K17 immunostaining did not reveal any keratin 17 expression in cultured NPFSCs (Figure S3A [see online supplementary material for a color version of this figure] purple). This result is consistent with our *in vivo* observations, where undifferentiated Tom-positive NPFSCs localized within the NPF lacked K17 expression (Figure 1E3, red population without green fluorescence), in contrast to K17-positive undifferentiated NSCs in the nail matrix (Figure 1E3, green fluorescence in pMx). However, increased calcium concentration to 0.3 mM induced the formation of more complex 2D structures, in which, some cells began to express K17 within 3 days, indicating their maturation to an NSC-like state (Figure S3A′ [see online supplementary material for a color version of this figure], purple). Notably, a subset of these cells also sporadically expressed the hard keratin marker AE13 under increased calcium conditions, marking the onset of terminal differentiation (Figure 6C′ and Figure S3B′ [see online supplementary material for a color version of this figure], purple), a feature absent in low-calcium cultures (Figure S3B [see online supplementary material for a color version of this figure]). In some differentiating cell clusters, the re-expression of the K15-GFP was sporadically observed (Figure 6C′ and Figure S3B′ [see online supplementary material for a color version of this figure], green). Within these structures, AE13-positive cells coexisted with K15-GFP-positive cells, suggesting that while some cells progressed toward terminal differentiation (AE13-positive, K15-GFP-negative or low), others may have re-entered a more quiescent, stem-like state (AE13-negative, K15-GFP-positive) (Figure 6C′, K15-GFP in green and AE13 in purple, detected in distinct zones of the same structure).

The 3D culturing was performed using Gri3D^®^ plates.^35^ Spheroid formation was observed within 5 days in low-calcium E-media (Figure 6C″, dashed outline). Within these spheroids, occasional K17 expression was detected (Figure 6C″, purple) and sporadic re-expression of K15-GFP in their central regions (Figure 6C″, green).

### BMP4 promotes NPFSC differentiation *in vitro*

To test whether BMP signaling influences NPFSCs’ behavior, cells were cultured under conditions differing in calcium concentration (low Ca^2+^ vs. medium Ca^2+^) and supplemented with increasing concentrations of recombinant BMP4 (0 ng/mL—blue arrow, 1 ng/mL—yellow arrow, or 10 ng/mL—orange arrow) (Figure 6D). To confirm activation of the BMP signaling pathway, phosphorylation of SMAD1/5 was assessed 2 hours after BMP4 supplementation. A dose-dependent increase in pSMAD1/5 immunofluorescence intensity was observed with increasing BMP4 concentrations, confirming rapid activation of canonical BMP signaling in NPFSCs (Figure 6E).

Next, the effect of the BMP4 on colony formation potential was evaluated. As expected the NPFSCs cultured in med Ca^2+^ media formed a higher number of colonies compared to low Ca^2+^ conditions across all BMP4 concentrations (Figure 6F). While BMP4 had no substantial effect on colony number, colony size was significantly influenced by BMP4 treatment, with larger colonies observed particularly at intermediate BMP4 concentrations (Figure 6G).

To further assess the impact of BMP4 on NPFSC differentiation, the expression of stem cell marker K15, a transition indicator for primed stem cells K17, and a differentiation marker AE13 were quantified by immunofluorescence. K15 expression remained relatively stable in low Ca^2+^ media but decreased under med Ca^2+^ environment with increasing BMP4 concentration (Figure 6H). In contrast, the expression of K17 and AE13 increased in response to BMP4 in med Ca^2+^ cultures (Figure 6I and J, respectively). K17 levels progressively increased with BMP4 supplementation (Figure 6I), while AE13 expression was strongly induced at higher BMP4 concentrations (Figure 6J). Representative images in Figure 6K show 20× magnification of cells expressing K17 and AE13 in med Ca^2+^ media in the absence (0 ng) or presence (10 ng) of BMP4. Between these two conditions, the most notable differences were observed in the morphology of cultured NPFSCs, which were significantly more flattened in high BMP4 concentration and started forming larger attached colonies with intense K17 and AE13 accumulation. Interestingly, in differentiation-promoting conditions, induced by elevated calcium alone, NPFSCs showed cytoplasmic AE13 signal within individual cells that retained clearly visible DAPI-positive nuclei (Figure 6K, middle right panel pointed by blue arrow: dapi—blue nuclei; purple—AE13), consistent with early stages of epithelial differentiation. In contrast, supplementation with high levels of BMP4 resulted in the formation of large, compact AE13-positive keratinized aggregates characterized by intense accumulation of hard keratin filaments. Notably, these structures frequently lacked detectable nuclear staining, suggesting progression to advanced stages of terminal differentiation toward a NP-like keratinocytes (Figure 6K, right panel pointed by orange arrow: dapi—blue nuclei; purple—AE13). Together, these findings suggest that BMP signaling not only promotes differentiation of NPFSCs but also facilitates the progression toward a terminal nail keratinization program and can be observed not only *in vivo* but also reproduced *in vitro*.

## Discussion

Mammalian digit tips exhibit limited intrinsic regenerative capacity, confined in their nail mini-organs, however, it is important to state that substantial anatomical and regenerative differences exist between mouse and human nails.^4^^,^^6^ While in mice, distal digit amputation preserves a Wnt-active nail matrix and enables robust regeneration of both the NP and the underlying P3, the human nail unit is structurally larger and flatter, with a more prominent proximal matrix, thus traumatic injuries rarely result in spontaneous regeneration, but rather fibrotic repair. Despite these differences, several key features are conserved between species, including the presence of nail-associated stem/progenitor cells in the NPF, expression of keratin markers associated with stemness and differentiation and the involvement of BMP and Wnt signaling pathways in nail growth and epithelial fate decisions.^4^^,^^6^ Thus, the murine nail unit remains a valuable model for identifying conserved cellular and molecular mechanisms that support nail-associated regeneration, and while findings of this paper do not imply direct phenotypic equivalence between species, they do define principles that may be relevant for improving regenerative responses in human nail and digit injury.

### NPFSCs contribute to the regeneration of the digit after distal permissive amputation

Using lineage tracing mouse model,^5^^,^^6^^,^^9^ we show that NPFSCs, arranged in a ring-like formation in the NPF (Figure 1B), contribute long-term to the peri-nail epidermis under homeostatic condition (Figure 1E1 arrow), but upon permissive distal amputation (Figures 1D and D′) can generate progeny to the regenerating nail matrix and contribute to NP reconstruction (Figure 1F1). In contrast, after non-permissive proximal amputations, delivery of NPFSC progeny to the matrix is markedly reduced (Figure 1H and H′), indicating that their regenerative contribution depends on preservation of a supportive nail matrix niche, associated with epithelial Wnt activity.^8^ In the regenerative distal amputations, Wntless and Lef1 (Figure 1F4,5) were maintained in the distal matrix, together with active BMP signaling, similarly as observed in uninjured lineage tracing control mice during their homeostatic growth (Figure 1E4,5). By contrast, non-regenerative proximal amputation lacked Wntless and showed reduced BMP activity (Figure 1H′), despite partial persistence of Lef1, suggesting that preservation of the distal matrix signaling environment is critical for efficient recruitment and differentiation of NPFSC-derived progeny. These findings are consistent with prior work demonstrating that epithelial Wnt signaling is essential for nail growth and digit regeneration.^8^^,^^18^^,^^36^

### Impaired digit regeneration in the absence of BMP signaling in nail epithelium

The inducible BMP LoF mice models enabled us to ablate the BMP signaling in the entire K14-expressing nail epithelial cells shortly after birth, thus allowing us to fully analyze their regenerative capabilities in adult life. Lack of BMP signaling, confirmed by inactivation of pSmad 1/5 and pSmad 1/5/9 (Figure 2D6,7 and E_6,7_), significantly impaired NP regeneration (Figure 2C1 and G orange plot) and blocked P3 restoration even after permissive distal amputation (Figure S1A‴ [see online supplementary material for a color version of this figure]), and was associated with loss of Wntless (Figure 2D4 and E4), marked reduction of Lef1 (Figure 2D5 and E5), and epidermalization of the nail unit (Figure 2A-C). Because this inducible BMP LoF model targets the broader nail epithelium rather than NPFSCs alone, our results indicate that BMP activity is required to maintain a regeneration-permissive epithelial state across the nail organ. In this context, the expansion of K15 expression (Figure 2E2) together with reduced proliferation (Figure 2E1) suggests that, in the absence of BMP signaling, matrix progenitors may shift toward a more quiescent, NPFSC-like state, while failing to execute proper nail differentiation.^9^

### NPFSCs participate in enhanced digit regeneration in the presence of constitutively active BMP signaling

The constitutive activation of BMP signaling in our BMP GoF mouse model was associated with accelerated NP regrowth following distal amputation (Figure 3K) and markedly improved P3 restoration, which fully regenerated and regained its native morphology within just 5 weeks PA (Figure S1A″ [see online supplementary material for a color version of this figure]). Moreover, this BMP activation shifted the regenerative boundary proximally, enabling complete regeneration following amputations extending to approximately 60% of P3 (Figure 3L and Figure S1C [see online supplementary material for a color version of this figure]). Interestingly, when a BMP GoF digit was subjected to an even deeper resection (extreme proximal amputation), that removed between 60% and 90% of the P3 along with most of the Wnt-active distal matrix, BMP continuous activation still supported the regeneration of the NP (Figure 3F-H′ and Figure S1D [see online supplementary material for a color version of this figure]). Notably, marked NPFSC progeny was observed to intensively replenish severely damaged nail matrix and missing NP after the extreme proximal amputation, participating at a much greater extent than in the case of any amputations performed distally (Figure 3I1-5 vs. J^1–5^), indicating that elevated BMP signaling can expand the functional contribution of this stem cell population after severe injury.

In the BMP GoF model, the Wnt signaling, indicated by Wntless protein expression, was active in the distal part of the restored nail matrix regardless of the injury (Figure 3I2 and J_2_), resembling its physiological localization in lineage tracing models (Figure 1E4 and F_4_). However, following an extreme proximal amputation, this relatively normal expression of Wntless in BMP GoF model (Figure 3I2) correlated with lack of signal from Lef1 (Figure 3I3) and significant reduction of β-catenin (Figure 3I5). These observations did not correlate with distal amputation, where expression of Wntless along with Lef1 was still present in distal nail matrix, with intense signal from β-catenin (Figure 3J2, J3, and J_5_, respectively). This expression pattern of Wnt signaling components suggests, that partial NP regeneration is still possible after the extreme proximal amputation, despite the absence of Lef1, as long as Wntless in maintained in the distal matrix. In this context, the proximal nail matrix, enriched in pSmads-positive cells due to BMP overexpression (Figure 3I4), appears to support the formation of a partially mature nail matrix with Wntless expression, likely through the active participation of NPFSC progenitors (Figure 3I1-5). These observations underscore the sufficiency of NPFSCs in driving regenerative processes in the presence of properly modulated BMP signaling, challenging the notion that only distal nail components are essential for regeneration and opening venues for therapeutic strategies targeting NPFSCs to enhance regeneration in non-regenerative injuries.

### BMP-Wnt signaling interplay in the digit restoration

Our observations suggest that BMP and Wnt pathways active in the nail mini-organ are largely dependent and partially arranged in a linear hierarchy. Genetic BMP ablation suppressed Wntless and strongly reduced Lef1 (Figure 2D4,5 and E_4,5_), indicating that BMP signaling is required to maintain key components of the epithelial Wnt signaling machinery. Conversely, ectopic activation of BMP signaling in nail epithelium, preserved or re-established Wntless expression in the remaining proximal matrix even after the extreme proximal amputations (Figure 3I2), despite the absence of Lef1 (Figure 3I3) and reduced β-catenin levels (Figure 3I5). This supports a model in which BMP signaling acts upstream of Wnt signaling as a permissive regulator that allows direct and at least partial activation of canonical Wnt signaling. While full activation of the canonical Wnt pathway (marked by Wntless, Lef1 and β-catenin) remains essential for complete digit regeneration, maintenance of Wntless expression alone, in the presence of constitutively active BMP signaling, is sufficient to support at least partial NP and digit regeneration driven by NPFSC progeny (Figure 3F, G, and I).

### Harvested NPF and cultured NPFSCs contribute to nail regrowth and digit regeneration upon transplantation

Finally, our *in vitro* studies and transplantation data provide proof that NPFSCs can be isolated from adult tissue, expanded in culture (Figure 5A and B), and remain functionally competent following engraftment. Cultured NPFSCs responded rapidly to BMP4 (Figure 6E), retained migration and colony-forming capacity (Figure 6A and B), and under differentiation-promoting conditions expressed markers associated with nail epithelial maturation and fate change: K17 (Figure S3A′ [see online supplementary material for a color version of this figure])—characteristic of vastly proliferating NSCs in the nail matrix, and AE13 (Figure S3B′ [see online supplementary material for a color version of this figure])—associated with NP formation. Simultaneous sporadic re-expression of K15-GFP in differentiating cultures, might suggest that while some cells undergo terminal differentiation, others may revert to a quiescent-like state (Figure 6C′). The BMP4 supplementation reduced the expression of stem cell marker K15 in differentiating NPFSCs (Figure 6H), while also significantly promoted the expression of K17 (Figure 6I) and AE13 (Figure 6J). Interestingly, increased calcium alone primarily prompted AE13 expression in nucleated cells, whereas strong BMP4 signaling induced large, anucleate AE13-positive keratinized aggregates (Figure 6K, far right panel indicated by orange arrow). These observations suggest that BMP signaling is required not merely to initiate differentiation-associated keratin expression but also to drive NPFSCs progression toward a terminal nail keratinization program. This *in vitro* phenotype mirrors key aspects of NP formation *in vivo* and supports a model in which BMP activity plays a key role in this process.

*In vitro*-expanded labeled NPFSCs that were subsequently harvested and directly transplanted into distally amputated digit tips of immunocompromised mice (Figure 5D) were observed to integrate into their native niche and contribute to nail regrowth (Figure 5E). Although the regenerated claw exhibited an unusually enlarged morphology (Figure 5D′), at this time point, we have not observed overt P3 regeneration, suggesting that the regenerating tip was still undergoing structural remodeling and osteogenic processes following histolysis. Strikingly, within this reorganizing extracellular nail matrix, Tom-positive NPFSCs progeny was also identified (Figure 5D″, asterisk). While these findings raise the intriguing possibility that NPFSCs might contribute to broader regenerative processes beyond the nail unit, including different digit tip compartments, further studies are required to determine whether their presence within the center of the regenerating digit reflects their transient plasticity or merely mechanical displacement during transplantation and regeneration. Interestingly, the positional plasticity of the NPFSCs was identified in subsequent experiment, in which an intact NPF tissue from BMP GoF mice was transplanted into the digits of immunocompromised mice with removed by plucking NP. Labeled NPFSCs, instead of remaining within their native niche, integrated into the distal nail bed of the regenerating digit from where they further contributed to NP elongation (Figure 4D and E). Collectively, our findings show that NPFSCs can be isolated from adult tissue, expanded *in vitro*, and still retain their stem cell properties and regenerative potential after transplantation, and together with the positional plasticity documented here, these features highlight NPFSCs as a promising cell source for future regenerative strategies targeting severe nail and digit injuries.

## Conclusions and future perspectives

In summary, our study identifies NPFSCs as previously underappreciated contributors to nail and digit tip regeneration and demonstrates that their activity is strongly shaped by BMP-Wnt signaling interactions within the nail epithelium. Enhanced activation of BMP signaling promotes a regeneration-permissive state, supports nail differentiation, accelerates P3 restoration, and expands regenerative capacity beyond previously established boundary, whereas loss of BMP disrupts both Wnt signaling and regeneration. BMP signaling governs the balance between stem cell maintenance and differentiation, what was supported by our *in vitro* analyses, which demonstrated that NPFSCs can be isolated from adult tissue and expanded in culture, where exogenous BMP stimulation promotes their differentiation toward nail epithelial fate. Importantly, we have evidenced that cultured NPFSCs retained their stem cell properties following transplantation, as the injected cells were capable of integrating into the native NPF niche and contributing to nail regrowth in injured digits. The regenerative competence and plasticity of NPFSCs therefore raise the possibility that these cells could be harnessed therapeutically to enhance nail and digit repair following traumatic injuries. In this context, BMP signaling may represent a key molecular cue regulating the activation and differentiation of NPFSCs during regenerative responses. Accordingly, strategies aiming to activate endogenous NPFSCs or transplant *ex vivo* expanded cells may represent promising avenues for regenerative medicine targeting the human nail unit and distal limb tissues.

## Supplementary Material

sxag028_Supplementary_Data

## Data Availability

Not applicable
