# Supplementary material for: Nail proximal fold stem cells participate in nail growth, orchestrating enhanced digit regeneration via bone morphogenetic protein signaling activation
Source: Stem Cells. 2026 Apr 8;44(8):sxag028. doi: 10.1093/stmcls/sxag028 (PMC13401488; doi:10.1093/stmcls/sxag028)

SUPPLEMENTARY INFORMATION

Nail Proximal Fold Stem Cells (NPFSCs) Participate In Nail Growth, Orchestrating Enhanced Digit Regeneration via BMP Signaling Activation

Running head: **NPFSCs Participate in Digit Regeneration via BMP**

Anna Pulawska-Czub[1], Alicja Olczak-Cossu[1] , Tomasz D. Pieczonka[1] , and Krzysztof Kobielak[1,2]*

Centre of New Technologies (CeNT), University of Warsaw (UW); 02-097 Warsaw, Poland; [2]Faculty of Medicine, University of Warsaw, 02-089 Warsaw, Poland

*Correspondence: k.kobielak@cent.uw.edu.pl tel: (+48) 22 55 43 731; Laboratory of Stem Cells, Development and Tissue Regeneration, Centre of New Technologies (CeNT), University of Warsaw (UW) 02-097 Warsaw, Poland

Supplementary Material and Methods

Animals

All animal studies were approved by the First Local Ethics Committee: No. 971/2020 as of 28 January 2020. The studies were conducted in accordance with the local legislation and institutional requirements. All mice were housed and bred within the animal facility at the Central Laboratory of Experimental Animals at the Medical University of Warsaw and at the Animal Facility at the University of Warsaw. The following mouse stocks were obtained from the Jackson Laboratory: **K15-GFP** B6.Cg-Tg(Krt1-15-EGFP)2Cot/J [1]; **tdTomato** B6.Cg-Gt(ROSA)26Sortm9(CAG-tdTomato)Hze/J; **K14-Cre-ER^{T2}** B6.Cg-Tg (KRT14-cre/ERT2)1Ipc/MtzJ [2]; **NUDE** B6.Cg-Foxn1nu/J; **K14rtTA** FVB-Tg(KRT14-rtTA)F42Efu/J [3]; **K15CrePR** B6;SJL-Tg(Krt1-15-cre/PGR*)22Cot/J [1]; **BMPR1A flox/flox** were a kind gift from Dr. Yuji Mishina [4,5]; **Tre-Alk3-Q233D (BMPR1A-CA)** was generated by Kobielak's lab [6]. By intercrossing the animals listed above, a following experimental lines were generated:
Lineage tracing murine model; "control model" – K15CrePR/Rosa26-STOP-tdTomato/K15GFP mice carrying a tdTomato (Tom) reporter gene with a floxed upstream stop cassette at the Rosa26 locus. Each paw of the experimental mouse was dipped in Ru468 stock solution (25mg/ml in 100%EtOH) for 3 seconds for five consecutive days at least a week prior to amputation and tissue collection. Following treatment, the stop cassette was excised, making Tom detectable in K15-expressing cells and their progeny. K15-GFP expression allows for cross-checking the location of these cells in their quiescent stage.
BMP GoF murine model – K15CrePR/Rosa26-STOP-tdTomato/K14rtTA/Tre-Alk3Q233 mice had the same system as the lineage tracing model and were induced with Ru468 in a similar manner to specifically label NPFSCs. The constitutively active form of BMP receptor type A1 was induced under a Doxy-inducible promoter [6]. The Doxy-supplemented diet was introduced to the parents of experimental mice on the day they were housed together for mating.
BMP LoF mice – K14CreERT/BMPR1A flox/flox mice were subjected to specific deletion of the BMP receptor type A1 in the entire K14-expressing nail epithelial cells prior to experiments by administering Tamoxifen (Sigma Aldrich #T5648) to pups from postnatal day 2 to day 6 (p2–p6). Each paw was dipped in Tamoxifen stock solution (12mg/ml in 100% EtOH) for 3 seconds for 5 consecutive days.

Digit tips amputations

Distal and proximal amputations were performed in mice anesthetized with isoflurane before amputations. The tip of one digit per paw was amputated. Buprenorphine was given at 0.1 mg/kg via subcutaneous injections for 5 days PA. Both types of amputations were performed in such a way that NPF with inherent NPFSCs remained intact in the paw. The regeneration process was observed for 3, 5, or 9 weeks. After this time the mice were sacrificed and all paws were collected and photographed using a LeicaM2 16 FA microdissection microscope. The length and area of each regenerated digit were analyzed in comparison to the corresponding amputated tip and to the neighboring, uninjured digit of the same paw. All measurements were made by Las X Leica Microsystems Software and ImageJ 1.54g software.

Digit tips or full paws were fixed in 4% paraformaldehyde for 24h at 4°C. They were rinsed in PBS 3 times and placed in 0,5M EDTA at pH 7.0 for 10 days at RT for decalcification. For prolonged storage, tissues were stored in a 30% sucrose gradient in 4°C. Prior to snap-freezing on the dry ice, tissues were embedded in O.C.T. FSC 22 blue (REF3801481 Leica) in vinyl cryomold tissue-tek (REF 4565 Sakura). Samples were further cut on Leica CM1860 cryostat to a thickness of 15 μm, mounted on SuperFrost Plus microscope slides (cat 631-0108 VWR), air dried at RT for 30 min and stored at -80°C. Before H&E or immunofluorescence staining, sections were defrosted and air dried for 10 min at RT.

Cell injections and tissue transplantations

NPFSCs were harvested and counted – around 1300 cells (viability over 90%; passages between 8-14) in one dose of 1μl low calcium (0.05mM Ca^{2+}) E-media were injected underneath the NPF of the 3rd (middle) finger of each paw of the anesthetized immunocompromised mice (NUDE). The same dose was injected into the 4th finger of the same paw that was subjected to distal amputation (removal only of the visible NP). The growth of digits was observed for 3 weeks. The animal was then sacrificed, and the full paws were collected and processed as described above.

Prior to BMP GoF NPF transplant, the receiver - immunocompromised NUDE mice, was placed on a Doxy-enriched diet. NPF tissue of the sacrificed BMP GoF mouse was collected using a fluorescent-guided microdissection microscope (LeicaM2 16 FA). Only dissected NPF tissues that were confirmed to harbor labelled K15-positive and Tom-positive cells were selected for the transplants and stored in PBS with antibiotic and antimycotic for up to 30 min prior transplantation. One nail per paw of anesthetized immunocompromised NUDE mice was removed by plucking, creating a pocket beneath the NPF for the tissue transplant. The regrowth of the NP was observed for 3 weeks, after which the digits were collected and processed as described above.

Hematoxylin and eosin staining and analysis

Cryosections were washed with PBS for 15 min, incubated in hematoxylin for 2 min, then washed in distilled water and left to dry. Subsequently, sections were incubated in an eosin mix for 1 min and washed in 95% ethanol. The dry sections were mounted in 80% glycerol. Images were made using an inverted phase contrast microscope Eclipse TS100 (Nikon, Japan). The area of P3 in uninjured digits and in amputated tips was measured in cross sections taken of the middle line of the digit using Image J 1.54g software.

Immunostaining

Cryosections were washed with PBS for 15 min and blocked in buffer containing 2% NGS, 2% BSA, and 0.5% Triton X-100 in PBS for 1 h at RT. Sections were incubated with primary antibodies overnight at 4°C. Antibodies were diluted in blocking buffer (2% BSA, 2% NGS in PBS) at the following dilutions: **AE13** (1:200 Abcam #ab16113), **β-Catenin** (1:1000 #C7207), **GFP** (1:500; Abcam #13970), **GPR177 (Wntless WLS** - 1:100 Invitrogen MA544978), **Ki67** (1:200; Abcam #ab15580), **K15** (1:200; Invitrogen #PA599461), **K17** (1:50; Invitrogen #MA5-31986) **Lef1 C12A5** (1:100; Cell Signaling Technology #2230) **Phospho–Smad1/5/9** (1:800; Cell Signaling Technology #13820), **Phospo–Smad1/5** (1:800; Cell Signaling Technology #9516). Then, sections were washed in PBS and incubated with secondary antibodies diluted in blocking buffer for 1 h at RT in the dark. Sections were thoroughly washed once in washing buffer (0,1% Triton X-100 in PBS) and two times in PBS, counterstained with a fluoromount containing 4′,6-diamidino-2-phenylindole (DAPI), and closed with cover slides. Prepared slides were stored at 4°C. Images were captured using a Zeiss LSM 700 confocal microscope or Zeiss Axis Observer fluorescent microscope and BC43 Ultimate Benchtop Confocal Microscope; Oxford Instruments.

NPFSCs isolation and *in vitro* culture

E-media [7] consisted of DMEM/F12 (3:1; Gibco, #90-5010SA) supplemented with 15% calcium-depleted fetal bovine serum (HyClone, #SH30071.03), 5 μg/mL insulin (Sigma-Aldrich, #I5500), 5 μg/mL transferrin (Sigma-Aldrich, #T2252), 0.5 μg/mL hydrocortisone (Merck, #31719), 100 U/mL penicillin and streptomycin (Gibco, #15070-063), 4 nM triiodo-L-thyronine (Sigma-Aldrich, #T2752-100MG), 10^{-10} M cholera toxin (Sigma-Aldrich, #C8052-1MG) and 2 nM L-glutamine (Biowest, #PAO205P021). For E-media medium calcium (E-MC) the media was supplemented with 0.3 mM $CaCl_2$ (Chempur, #118748709) or 0.05 mM $CaCl_2$ for low calcium E-media (E-LC).

Nail upper epidermis covering the base of the NP in the lineage tracing mouse models was tilted back in order to reveal K15-GFP-positive quiescent NPFSCs in the NPF. Labelled tissue was collected using a fluorescent-guided microdissection microscope (LeicaM2 16 FA). Dissected NPF tissue was mechanically dissociated in PBS with antibiotic and antimycotic and then enzymatically digested for 1h in 0,25% Trypsin (Biowest, #L0931-100) at 37°C prior to seeding the unrestricted cells into coated with collagen (3h in 37°C) and fibronectin (1h in 37°C) Petri dish containing stem cell-supportive culture medium E-MC. *In vitro* cell cultures were maintained in an incubator at a temperature of 37°C, 5% CO_2 concentration, and controlled humidity. Cells labeled with green and red fluorescent proteins were further manually separated and cultured in order to expand and establish a pure NPFSC cell line. Cell homogeneity was assessed by flow cytometry on a FACSAria Fusion (BD Biosciences), confirming that the entire population was Tom-positive. E-MC was switched to E-LC media after the third passage.

Colony formation assay

A total of 2,000 NPFSCs (n = 3) or hfSCs (n = 3) were seeded into each well of a 6-well plate and cultured in the E-LC medium. Cultures were terminated after 5 days, and cells were fixed with cold 100% methanol for 10 min, followed by 4% PFA for 15 min at RT. Cell colonies were stained with 0.01% crystal violet solution. The results were visualized with Bio-Rad Molecular Imager GelDock XR+, and the colony size, as well as colony number, were counted using ImageJ software.

Wound healing *in vitro* assay

NPFSCs or hfSCs were cultured *in vitro* until they reached 100% confluence. Subsequently, they were incubated with mitomycin C (final solution 8 μg/mL) for 2 h. After incubation, the medium was removed, and using sterile 10 μL pipette tips, two perpendicular scratches were made across the bottoms. The plate was incubated in the incubator of the BC43 Ultimate Benchtop Confocal Microscope; Oxford Instruments, at 37°C and 5% CO_2, and images of cell cultures were captured at 3 h intervals for 3 days or until the cells completely covered the scratches. ImageJ software was utilized for further analysis of the captured images.

Cell differentiation

Sterile round glass coverslips (12 mm diameter) were pre-placed at the bottom of each well of a 12-well tissue culture plate. NPFSCs were seeded at a density of 4,000 cells per well, and hfSCs – 2,000 cells per well (due to their higher proliferation potential). Cells were incubated in E-LC media for 2 days under standard conditions (37°C, 5% CO_2). Medium was changed to E-MC on the 3rd day, and the incubation was continued for the next 3 days. Next, cells were washed with PBS and fixed with 4% PFA for 15 min at 37°C. The immunofluorescent staining was performed on the fixed cells as described in the previous methods. For undifferentiating control, the E-LC media on the 3rd day was replaced with the same E-LC media.

BMP4 supplementation

Recombinant human BMP-4 protein (Qkine; Qk038) was reconstituted according to the manufacturer's instructions by dissolving the lyophilized product in 10mM HCl to obtain a stock concentration of 50 μg/ml. The appropriate volume of BMP-4 stock solution was added to either E-LC or E-MC culture media to achieve final concentrations of 1 ng/ml or 10 ng/ml of BMP-4 per ml of media. Control conditions (0ng/ml) received an equivalent volume of 10mM HCl (vehicle) without BMP-4. Immunofluorescence staining was performed as described above. Fluorescence intensity was quantified using ImageJ software (NIH), and statistical analysis of differences between conditions was carried out using GraphPad Prism (GraphPad Software).

NPFSCs spheroid formation

Spheroids collected from Gri3D® well plates (Sun Bioscience, Sample Plate 400-800 μm) were prepared in accordance to the manufacturer's instructions [8]. The seeded NPFSCs at 14th passage started to form spheroids on the 5th day in E-LC medium; on the 11th day they were collected for immunostaining as described previously.

Statistical analysis

Statistical analyses were carried out using the Prism software package (GraphPad). The significance of differences between two groups was determined using the unpaired, two-tailed Student's t test. Analyses of multiple groups were performed using One- Way ANOVA. Statistical significance was denoted by asterisks ($P < 0.1$ [*], $P < 0.01$ [**], and $P < 0.001$ [***]. The data are presented as mean ± SEM.

1 Morris RJ, Liu Y, Marles L et al. Capturing and profiling adult hair follicle stem cells [in eng]. Nat Biotechnol 2004;22(4):411-417.

2 Vasioukhin V, Degenstein L, Wise B et al. The magical touch: genome targeting in epidermal stem cells induced by tamoxifen application to mouse skin [in eng]. Proc Natl Acad Sci U S A 1999;96(15):8551-8556.

3 Nguyen H, Rendl M, Fuchs E. Tcf3 governs stem cell features and represses cell fate determination in skin [in eng]. Cell 2006;127(1):171-183.

4 Kobielak K, Pasolli HA, Alonso L et al. Defining BMP functions in the hair follicle by conditional ablation of BMP receptor IA [in eng]. J Cell Biol 2003;163(3):609-623.

5 Mishina Y, Hanks MC, Miura S et al. Generation of Bmpr/Alk3 conditional knockout mice [in eng]. Genesis 2002;32(2):69-72.

6 Kandyba E, Leung Y, Chen YB et al. Competitive balance of intrabulge BMP/Wnt signaling reveals a robust gene network ruling stem cell homeostasis and cyclic activation [in eng]. Proc Natl Acad Sci U S A 2013;110(4):1351-1356.

7 Rheinwald JG, Green H. Epidermal growth factor and the multiplication of cultured human epidermal keratinocytes [in eng]. Nature 1977;265(5593):421-424.

8 Brandenberg N, Hoehnel S, Kuttler F et al. High-throughput automated organoid culture via stem-cell aggregation in microcavity arrays. Nature Biomedical Engineering 2020;4(9):863-874.

Supplementary Figure S1. Nail mini-organ injury model involving amputations at different levels along the P3. **(A)** H&E staining of uninjured digits (far left), distally amputated tips (middle left), and regenerating digits at 5 weeks (middle right) and 9 weeks (far right) after distal amputation in control – lineage tracing (**A'** top row), BMP GoF (**A''** middle row), and BMP LoF (**A'''** bottom row) mouse models. **(B)** Scatter plot showing the percentage of digit regeneration (y-axis) relative to the percentage of P3 volume amputated (x-axis) in control model digits (blue dot for each regenerating digit). The red dashed line marks the approximate boundary between distal and proximal amputations (~50% P3 removal), which in control mice models defines the transition between regenerative and non-regenerative outcomes (orange dashed line). The green arrow indicates the direction of progressing digit regeneration, with distal amputations supporting full or even over-growth of the NP, while proximal amputations (> 50% P3 loss) prevent any regeneration. **(C)** Comparison of P3 resection extent between control (blue) and BMP GoF (green) digits that still supported successful P3 regeneration. **(D)** Example of an extreme proximal amputation in a BMP GoF mouse (removal of 60–90 % of the P3) showing partial regeneration of the NP but no reconstruction of the P3. Scale bars, 500 µm

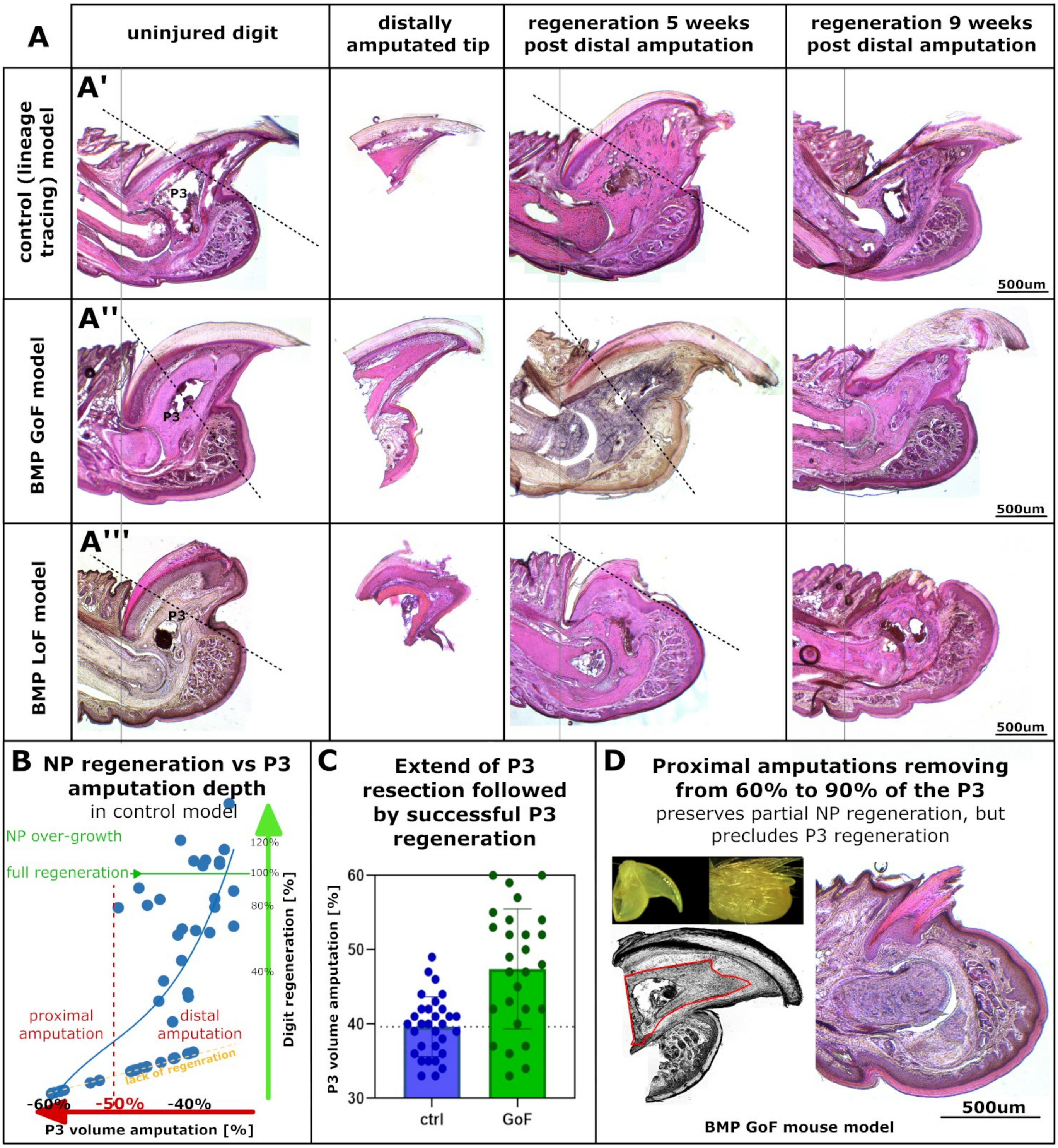

Supplementary Figure S2. Structural similarities between control (lineage tracing) model and BMP GoF digits. (A) *Immunostaining with cytokeratin K17 Ab.* ***(A_1)*** *K17 is observed in the proximal matrix of the uninjured digit in a similar manner to* ***(A_3)*** *BMP GoF model.* ***(A_2)*** *Following distal amputation, the K17 area expands to the regenerated distal matrix in both* ***(A_2)*** *control and (A4) BMP GoF model.* ***(B)*** *Immunostaining with Ki67 Ab.* ***(B_1)*** *Highest proliferation is observed in the proximal matrix of the* ***(B_1)*** *control and* ***(B_3)*** *BMP GoF model.* ***(B_2)*** *During regeneration, more proliferation is additionally observed in the distal region of the matrix in both* ***(B_2)*** *control and* ***(B_4)*** *BMP GoF model.* ***(B_3, B_4)*** *BMP GoF model consistently shows higher proliferation rate than* ***(B_1, B_2)*** *control model.* ***(C)*** *Immunostaining with Wntless and Lef1 Ab – markers of activated Wnt signaling.* ***(C_1)*** *Lef1 in the bottom distal matrix appears similar to* ***(C_5)*** *Wntless in uninjured control model digits. During regeneration* ***(C_2)*** *Lef1 and* ***(C_6)*** *Wntless are also found in the distal matrix.* ***(C_3)*** *Lef1 was identified in distal matrix in the uninjured BMP GoF, similarly to its location in the* ***(C_4)*** *regenerated digit, as well as that of Wntless in* ***(C_7)*** *uninjured and* ***(C_8)*** *regenerating digits, was similar to control model digits (*C_5*,* C_6*, respectively).* ***(D)*** *Immunostaining with pSmad1/5 and pSmad1/5/9 Ab – markers of activated BMP signaling.* ***(D_1)*** *pSmad1/5 is seen in the distal matrix of uninjured and* ***(D_2)*** *regenerating control model digits, while significantly higher levels appear in* ***(D_3, D_4)*** *BMP GoF digits, extending into proximal matrix.* ***(D_5)*** *pSmad1/5/9 is seen in the distal matrix of uninjured control model digits and* ***(D_6)*** *expands during regeneration.* ***(D_7)*** *In contrast, elevated Smad1/5/9 is found throughout the distal and proximal matrix floor in both* ***(D_7)*** *uninjured and* ***(D_8)*** *regenerating BMP GoF digits.*

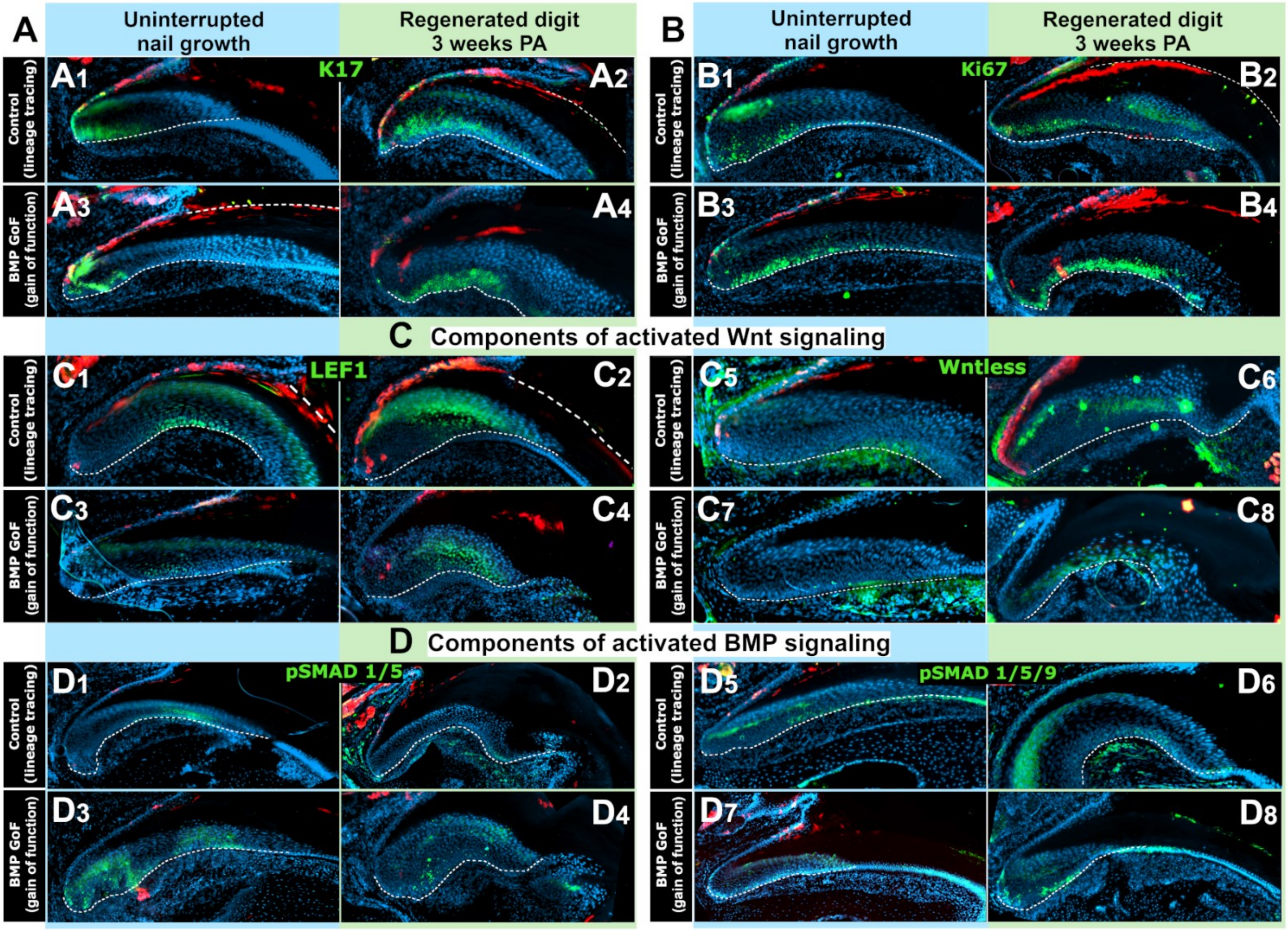

Supplementary Figure S3. NPFSCs differentiation. (A) K17 staining (purple) of the NPFSCs cultured for 5 days in low calcium E-media ($CaCl_2$ concentration 0.05 mM) in comparison to **(A')** NPFSCs cultured 2 days in low calcium E-media followed by 3 days in medium calcium E-media ($CaCl_2$ concertation 0.3 mM). **(B)** AE13 staining (purple) of the NPFSCs cultured for 5 days in low calcium E-media in comparison to **(B')** NPFSCs cultured 2 days in low calcium E-media followed by 3 days in medium calcium E-media.

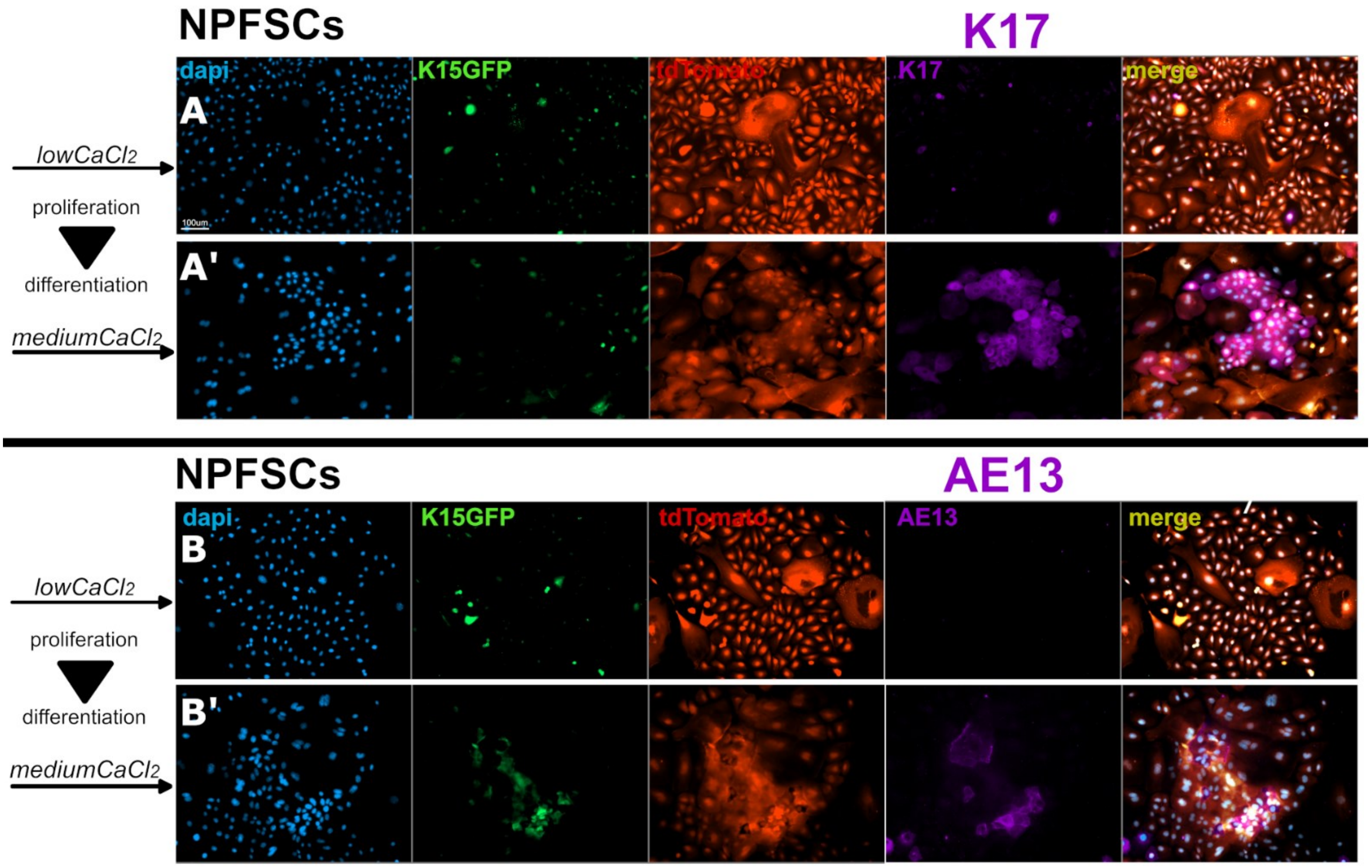

Supplement: sxag028_Supplementary_Data [file sxag028_supplementary_data.pdf]
